# Higher-Order Dimensions of Psychopathology in a Neurodevelopmental Transdiagnostic Sample

**DOI:** 10.1037/abn0000710

**Published:** 2021-11

**Authors:** Joni Holmes, Silvana Mareva, Marc P. Bennett, Melissa J. Black, Jacalyn Guy

**Affiliations:** 1 Medical Research Council Cognition and Brain Sciences Unit, University of Cambridge; 2 Cambridgeshire and Peterborough National Health Service Foundation Trust, Cambridge, United Kingdom

**Keywords:** neurodevelopmental, transdiagnostic, childhood psychopathology, general p factor, hierarchical dimensional model

## Abstract

Hierarchical dimensional models of psychopathology derived for adult and child community populations offer more informative and efficient methods for assessing and treating symptoms of mental ill health than traditional diagnostic approaches. It is not yet clear how many dimensions should be included in models for youth with neurodevelopmental conditions. The aim of this study was to delineate the hierarchical dimensional structure of psychopathology in a transdiagnostic sample of children and adolescents with learning-related problems, and to test the concurrent predictive value of the model for clinically, socially, and educationally relevant outcomes. A sample of *N* = 403 participants from the Centre for Attention Learning and Memory (CALM) cohort were included. Hierarchical factor analysis delineated dimensions of psychopathology from ratings on the Conner’s Parent Rating Short Form, the Revised Children’s Anxiety and Depression Scale, and the Strengths and Difficulties Questionnaire. A hierarchical structure with a general p factor at the apex, broad internalizing and broad externalizing spectra below, and three more specific factors (specific internalizing, social maladjustment, and neurodevelopmental) emerged. The p factor predicted all concurrently measured social, clinical, and educational outcomes, but the other dimensions provided incremental predictive value. The neurodevelopmental dimension, which captured symptoms of inattention, hyperactivity, and executive function and emerged from the higher-order externalizing factor, was the strongest predictor of learning. This suggests that in struggling learners, cognitive and affective behaviors may interact to influence learning outcomes.

## General Scientific Summary

This study identifies dimensions of psychopathology in a sample of children with neurodevelopmental difficulties who are at increased risk for mental health problems. Dimensions capturing neurodevelopmental concerns were the strongest predictors of the children’s current learning outcomes.

Our taxonomies of mental health difficulties have evolved rapidly over the past decade. This has been fueled both by a greater awareness of the challenges of category-based diagnostic nosology (Dalgleish et al., 2020) and the emergence of new methodological approaches that enable empirically derived frameworks to incorporate the complexity of signs, symptoms, and behaviors that characterize mental health struggles (Borsboom & Cramer, 2013; Goldberg, 2006). The traditional diagnostic rubric endorsed by international classification systems such as the *Diagnostic Statistical Manual of Mental Disorders-Fifth Edition* (*DSM–5*; American Psychiatric Association, 2013) defines mental disorders as distinct and discrete categories. This categorical approach runs counter to a wealth of clinical and research evidence showing that disorders are highly comorbid, heterogeneous, variable across development and the lifespan, explained by multiple causes, and not captured by a cardinal set of symptoms (Dalgleish et al., 2020). An alternative, dimensional, approach emphasizes the importance of continuous factors that span the full range of functioning, from adaptive to maladaptive, that can cut across traditional categories of mental ill health (e.g., Caspi et al., 2014; Caspi & Moffitt, 2018; Lahey et al., 2012, 2017; Martel et al., 2017; Patalay et al., 2015).

There are multiple dimensional models of psychopathology (e.g., Caspi et al., 2014; Kotov et al., 2017). Extant approaches, validated for typically developing young people and adults, may not be generalizable to those with neurodevelopmental conditions. This is important since those experiencing neurodevelopmental difficulties are at increased risk of poor mental health outcomes (e.g., Francis et al., 2019; Sciberras et al., 2014; Vaillancourt et al., 2017; Yew & O’Kearney, 2013). To date, very few attempts have been made to delineate symptom dimensions for those with neurodevelopmental conditions (although see Rodriguez-Seijas et al., 2020, for a dimensional account in autistic youth).

The primary aim of this study was to delineate higher-order dimensions of psychopathology in a neurodevelopmental transdiagnostic sample of children and adolescents with learning-related problems. Previous attempts to identify dimensions of psychopathology in at-risk children have included children with specific developmental disorders using diagnostic ratings scales, and traditional factor modeling approaches that distil distinctions between dimensions and symptoms (e.g., Rodriguez-Seijas et al., 2020 used confirmatory factor analytic tools on *DSM–IV* ratings with autistic children). In the current study, transdiagnostic measures spanning internalizing and externalizing symptoms and problems related to neurodevelopmental disorders were collected from a transdiagnostic sample. A data-driven approach was adopted that enabled the retention of shared variance between measures at each level of the hierarchy—an approach consistent with transdiagnostic goals to identify shared processes. As such, this study provides the first hierarchical transdiagnostic model of multiple transdiagnostic dimensions of symptoms related both to psychopathology and developmental difficulties in a transdiagnostic sample. A second aim of the study was to explore whether each level of the hierarchy differentially predicted children’s clinical, educational, and social outcomes.

## Dimensional Models of Psychopathology

Dimensional models of psychopathology account for widespread comorbidity between disorders. Early models assumed that two or three dimensions best explained the high rates of co-occurrence between disorders in both adults and children (e.g., Achenbach & Edelbrock, 1981; Kendler et al., 2003; Krueger, 1999; Wright et al., 2013). Considerable covariation between these early dimensions led to the development of contemporary frameworks that conceptualize psychopathology as multiple hierarchically organized transdiagnostic dimensions (e.g., Caspi et al., 2014; Kotov et al., 2017; Lahey et al., 2012; Michelini et al., 2019; Patalay et al., 2015). These frameworks (e.g., the Hierarchical Taxonomy of Psychopathology [HiTOP]; Kotov et al., 2017) include a general factor of psychopathology, which sits above spectra that align with broad internalizing and externalizing factors. These spectra then become progressively more specific, breaking down into lower-order dimensions that align with subsets of traditional diagnoses or disorders that tend to co-occur (Slade & Watson, 2006), and then into symptom components or individual symptoms as the lowest tier of the hierarchy.

## Hierarchical Integrative Frameworks

Traditional approaches for identifying the optimal number of dimensions in transdiagnostic hierarchical frameworks rely on factor analytic methods (e.g., exploratory factor analysis [EFA] or principal component analysis [PCA]) that extract variance in higher-order factors from the lower-order factors. Simply put, the variance explained by one factor in the top level of the hierarchy is split between the factors identified in the next tier of the hierarchy. The net result being that lower-order factors in traditional models capture subtle distinctions *between* indicators (e.g., what makes social phobia distinct from generalized anxiety disorder), and not shared aspects *within* factors (e.g., what makes the two disorders more similar). Identifying what separates or distinguishes symptoms or syndromes from one another is not congruent with a transdiagnostic approach.

Relatively new empirically derived multilevel hierarchical models can address this issue (e.g., Farmer et al., 2013; Forbes et al., 2017; Kim & Eaton, 2015). Goldberg’s (2006) bass-ackward factor analytic method enables the sequential extraction of dimensions from the top down: it extracts maximally distinct orthogonal components at each level of the hierarchy, starting with the extraction of a single component at the highest level, two at the second level, and so on. Crucially, it maps all measures in a multidimensional space (all indicators load on all components at each level of the hierarchy), and allows each level of the hierarchy to retain all the variance in the patterns of covariation among the symptoms. In retaining what is shared between indicators at each level of hierarchy, this approach is congruent with transdiagnostic approaches that aim to understand the *shared* mechanisms underlying symptoms that co-occur across traditional categorical disorders (e.g., Cuthbert & Insel, 2013; R.-Mercier et al., 2018; Newby et al., 2015; Owen, 2014; Reininghaus et al., 2019; Sakiris & Berle, 2019; Titov et al., 2011).

A few studies have applied Goldberg’s bass-ackward (Goldberg, 2006) approach to identify hierarchies of higher–order dimensions of predominantly personality pathologies in adult populations (e.g., the Alternative Model for Personality Disorders; Forbes et al., 2017; Morey et al., 2013; Tackett et al., 2008; Wright et al., 2012). To our knowledge, there has only been one attempt to apply this method to delineate only higher-order dimensions of psychopathology. Using data from the Adolescent Brain Cognitive Development study, Michelini et al. (2019) identified a hierarchical structure with a general psychopathology factor at the first level, and five specific factors (internalizing, externalizing, somatoform, detachment, and neurodevelopmental) in a community sample of children. Many of these specific factors were included in other HiTOP, with the exception of the neurodevelopmental dimension. Crucially, each level of the hierarchy identified by Michelini et al. (2019) differentially predicted different outcomes for the children. Notable findings were specific links between the p factor and the use of mental health services, and a strong relationship between the newly identified neurodevelopmental factor and children’s academic functioning. Michelini’s (2019) findings underscore the importance of including symptoms of neurodevelopmental disorders, such as inattention, in models of psychopathology, and demonstrate the validity of examining multiple levels of the hierarchy of psychopathology to characterize children’s mental health symptoms and their relevance to different aspects of clinical and educational functioning.

## The Current Study

The aim of this study was to use the bass-ackward method to delineate hierarchical dimensions of psychopathology in a cohort of children with learning-related problems, and to test the links between the emergent dimensions and children’s educational, social, and clinical functioning. This is because mental ill health and diminished psychosocial functioning are common among children with diagnosed neurodevelopmental disorders of learning. Multiple studies report elevated levels of internalizing symptoms among children with attention-deficit-hyperactivity disorder (ADHD) and autism spectrum disorder (ASD; e.g., Jarrett & Ollendick, 2008; Larson et al., 2011; Rodgers & Ofield, 2018; Sciberras et al., 2014; Vaillancourt et al., 2017). Externalizing symptoms can also feature among these groups, for example, substance misuse, aggressive behavior, or unsafe sex (Baker et al., 2018; Gillberg et al., 2004; Vaillancourt et al., 2017). Children with diagnoses of language and reading disorders also experience internalizing problems (e.g., Beitchman et al., 2001; Boetsch et al., 1996; Yew & O’Kearney, 2013), but externalizing difficulties are reported less often. A substantial proportion of children who have not received a diagnosis, but who nonetheless are struggling at school, also experience heightened levels of mental health problems (Arnold et al., 2005; Auerbach et al., 2008; Bryant et al., 2020; Francis et al., 2019; Greenham, 1999; Maughan & Carroll, 2006; Willcutt et al., 2013; Wu et al., 2014; Young et al., 2012).

Studies attempting to understand psychopathology in pediatric populations with learning-related problems typically adopt case-controlled designs that group individuals according to the presence or absence of one or more diagnosed neurodevelopmental conditions (e.g., Humphreys et al., 2012; Rodriguez-Seijas et al., 2020). However, like psychiatric disorders, neurodevelopmental conditions are characterized by high degrees of intracondition variation and intercondition commonality. This has motivated a shift toward more transdiagnostic approaches whereby individuals are characterized based on well-known cognitive (Holmes et al., 2020), behavioral (Mareva et al., 2019) and neurobiological (Siugzdaite et al., 2020) mechanisms as opposed to their diagnostic status. To date, there have been no attempts to delineate hierarchical dimensions of psychopathology in a neurodevelopmental transdiagnostic sample.

The present study recruited a highly heterogeneous pediatric sample with a range of neurodevelopmental symptoms linked to poor learning. It adopted a functionally defined approach of enrolling individuals identified by practitioners as having difficulties in attention, learning, and/or memory. These individuals did not fit traditional categories of neurodevelopmental disorders; some had a single diagnosis, others had multiple diagnoses, but the majority were undiagnosed despite coming to the attention of a health or educational professional for experiencing difficulties that were affecting their school progress. The sample included children with relatively mild problems, who would likely not meet diagnostic thresholds for specific learning disorders, in addition to many children whose more marked problems definitely would. A considerable proportion of the sample had a diagnosis of ADHD.

The hierarchical model was derived using subscales from three inventories: the Revised Child Anxiety and Depression Scale - Parent Version (RCADS; Chorpita et al., 2000), the Conners-3 Parent Short Form (CPSF; Conners, 2008) and the Strengths and Difficulties Questionnaire (SDQ; Goodman, 1997). These were selected for two reasons. First, they provide a broad sweep of internalizing and externalizing behaviors. Second, they are widely used in clinical practice to determine symptom severity, enhancing the translational relevance of our findings. The measures selected for inclusion maximize the breadth of the internalizing and externalizing symptoms while avoiding including very similar measures of the same symptom. Data reduction methods such as the one used here identify the main axes of variation in selected measures, representing the largest amounts of the variance within the dataset. Including multiple indicators of some symptoms and not others can influence the dimensions that emerge (for example, if three indicators of hyperactivity were included and only one of inattention, a “hyperactivity” dimension might emerge as distinct from inattention simply because the input was weighted more heavily toward capturing hyperactivity). To avoid this, a single indicator of each symptom was included from the subscales (see Method for details about subscale selection). Subscales from the SDQ and CPSF that were not included in the model were included as clinical outcomes alongside measures from the Behavior Rating Inventory of Executive Function (BRIEF; Gioia et al., 2000) to test whether levels of the hierarchy differentially predicted different aspects of clinical function. The Learning Problems subscale from the CPSF was held out of the model to test how the different levels of the hierarchy predicted concurrent education performance. This was of particular interest given the children were referred primarily for experiencing learning difficulties. There were no predictions about the specific dimensions that would emerge as the data analysis was exploratory. Nonetheless, a general p factor and two higher-level spectra (internalizing and externalizing) were expected given they are well-established dimensions of psychopathology in both traditional and newer hierarchical models of psychopathology (e.g., Caspi et al., 2014; Kotov et al., 2017; Michelini et al., 2019). We modeled the associations between the emergent dimensions and children’s learning, clinical, and social functioning. These analyses were also exploratory.

## Method

### Participants

A sample of *N* = 403 (*M*_age_ = 9.73, *SD* = 2.49; females, *N* = 125, *M*_age_ = 9.81, *SD* = 2.56; males, *N* = 278, *M*_age_ = 9.69, *SD* = 2.45) children was drawn from the Centre for Attention Learning and Memory (CALM) cohort. This included all participants for whom RCADS data were available. The wider CALM cohort includes 805 children tested between February 2014 and January 2019 (Holmes et al., 2019). The RCADS was introduced into the study protocol in December 2016, explaining why these data are only available for a subset of the wider cohort, and why only a subset of the wider sample is included in the current analysis.

The current sample included *N* = 212 children without a diagnosis (52.61%) and *N* = 191 (47.39%) with at least one diagnosis. *N* = 230 (57.07%) were referred by a professional working in education (e.g., a special-needs teacher), *N* = 164 (40.69%) were referred by a health practitioner (e.g., pediatrician), and *N* = 9 (2.23%) were referred by a Speech and Language Therapist. An Index of Multiple Deprivation (IMD; Ministry of Housing Communities & Local Government, 2020) classified the socioeconomic status of the sample. Scores for different local areas in the United Kingdom range from 1st to 32 844th (most to least deprived)· The range of IMD for the sample (see Table 1) indicated participants came from areas with varying degrees of deprivation, with an average ranking above the national median. The data analyzed include the parent-rated subscales of the RCADS, CPSF, SDQ, and BRIEF (Gioia et al., 2000).[Table tbl1]

### Materials and Procedure

Children aged 5 to 18 years were referred to CALM by health and education professionals for problems in attention, learning and/or memory. Children completed a 4-hr assessment of learning and cognition, and parents/legal guardians/carers completed questionnaires measuring the child’s behavior and mental health. All procedures complied with the ethical standards of the national and institutional committees on human experimentation and with the Helsinki Declaration of 1975, as revised in 2008. All procedures involving human participants were approved by the National Health Service (REC: 13/EE/0157). Parents/caregivers provided written consent and child verbal assent was obtained.

#### Psychopathology

Three measures used widely in clinical practice and capture internalizing and externalizing symptom severity were selected: the RCADS, CPSF, and SDQ. All six subscales from the RCADS were included in the hierarchical dimensional model: Separation anxiety disorder, Social phobia, Generalized anxiety disorder, Panic disorder, Obsessive–compulsive disorder, and Depression. The following subscales from the CPSF were also included: Inattention, Hyperactivity/impulsivity, Executive function, Aggression, and Peer relations. Two subscales from the SDQ were also included: Prosocial behavior (reverse coded to reflect low prosocial behavior) and Conduct problems. The measures selected for inclusion in the model were chosen to maximize the range of symptoms captured, while including only a single indicator of any particular symptom from all subscales. Including multiple indicators of some symptoms and not others can bias the dimensions that emerge. For this reason a single indicator of each symptom was selected. Subscales from the RCADS were chosen as the primary input to capture multiple symptoms of internalizing difficulties; this was preferable to using the Emotion subscale from the CPSF that combines symptoms of anxiety and depression into a single measure. Next, subscales from the CPSF that did not overlap with the RCADS were included to capture both externalizing symptoms and symptoms associated with neurodevelopmental difficulties. The CPSF was prioritized over the SDQ at this point as it captures more symptoms of neurodevelopmental problems. Subscales from the SDQ were then used to supplement the model with additional externalizing domains not captured by the RCADS and CPSF.

#### Predicted Outcomes

The predictive value of different levels of the model were estimated using a limited number of variables from the CALM dataset. Some of these assessed concurrent clinical outcomes: Emotional symptoms, Peer relationship problems, and Hyperactivity and Inattention from the SDQ, and the Global executive function composite from the BRIEF. Concurrent educational performance was measured using the Learning problems subscale from the CPSF. Finally, the IMD was used as a proxy for socioeconomic status.

### Analysis Plan

First, principal components analysis was used to extract and rotate (with geomin) factor solutions for the measures of psychopathology. The maximum number of factors to extract was determined by parallel analysis. All factor structures from one to the maximum number were considered. Second, the hierarchical structure was derived by correlating factor scores on adjacent levels of the hierarchy using Goldberg’s bass-ackward hierarchical method (Goldberg, 2006). This is the only available method to delineate multiple hierarchical levels using an exploratory approach. It allows factors to be correlated across levels without statistically removing variance shared with a general factor. To test the predictive value of the dimensions, factor scores from each level were entered into a series of regression models with the predicted outcomes as dependent variables. The incremental predictive value of each level of the hierarchy was examined by testing the significance of changes in *F* and *R*^2^ (Δ*F* and Δ*R*^2^) between models with different numbers of factors as predictors. All analyses were conducted in R Version 4.0.3 using the Psych package 2.0.12.

## Results

The sample profile is summarized in Table 1. Scores were in the age-typical range for Prosocial behavior and all subscales of RCADS, except Depression, which was borderline elevated. Aggression as rated on the CPFS, and Conduct problems, Emotional symptoms, and Peer relationship problems from the SDQ were also borderline elevated. Scores were in the clinical/abnormal range on the remaining CPSF subscales, Hyperactivity and Inattention (SDQ), and the Global executive composite (BRIEF).

### Hierarchical Structure of Psychopathology

**Figure 1 fig1:**
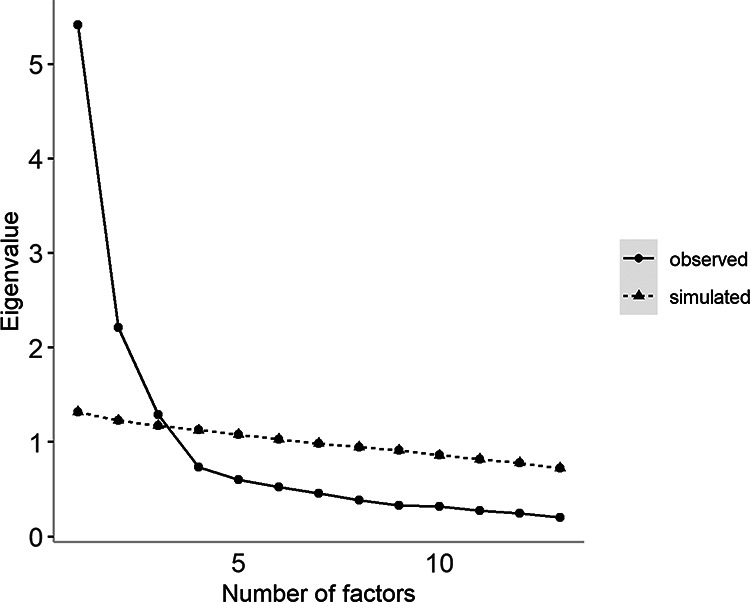
Parallel Analysis of the RCADS, CPSF, and SDQ Subscales *Note.* The simulated line is the top of the 95% confidence interval (CI) around the simulated eigenvalues. SDQ = Strengths and Difficulties Questionnaire; CPSF = Conners-3 Parent Rating Scale Short Form; RCADS = Revised Child and Anxiety and Depression Scale (Parent Version).

The maximum number of factors to extract was determined with parallel analyses (extraction was stopped when eigenvalues fell within the 95% confidence interval (CI) of eigenvalues from simulated data). This indicated that up to three factors could be extracted (see Figure 1). For completeness, a four-factor solution was also considered (see Supplemental Materials Table 8). This produced a model with a single indicator on the fourth factor, and eigenvalues that were outside the acceptable 95% CI. With fewer than three indicators on the fourth factor it was not possible to interpret (Fabrigar et al., 1999; Velicer & Fava, 1998), indicating the maximum number of factors that could be extracted was three.[Fig fig1]

**Figure 2 fig2:**
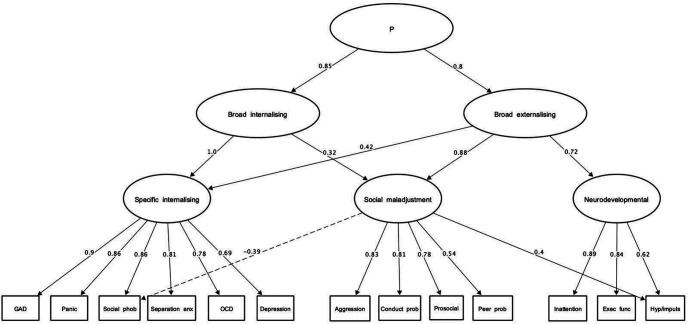
Hierarchical Structure of Psychopathology in the CALM Sample *Note.* Values represent correlations, according to Goldberg’s bass-ackwards hierarchical method. The dashed arrow represents a negative association between a latent dimension and observed variable. CALM = Centre for Attention Learning and Memory; GAD = General anxiety disorder; OCD = Obsessive Compulsive Disorder; Panic = Panic disorder; Social phob = Social phobia; Separation anx = Separation anxiety (all from RCADS); Conduct prob = Conduct problems; Prosocial = Prosocial behavior (reverse coded; SDQ); Peer prob = Peer relations; Exec func = Executive functions; Hyp/Impuls = Hyperactivity and impulsivity (all from CPSF). P = general psychopathology. Paths below .30 are omitted.

The factor loadings, extracted using principal components, are presented in Table 2. Note that although components were extracted, the term “factor” is used from here on for ease of interpretability and because the two terms are used interchangeably in studies adopting the same bass-ackward methods (e.g., Michelini et al., 2019). Also, the term factor is more synonymous with dimensional approaches in the wider literature. The one, two and three factor models were tenable and interpretable; see Figure 2 for the hierarchical structure.[Table tbl2][Fig fig2]

The one factor solution reflected a general psychopathology p factor, and the two factor solution broad internalizing and broad externalizing factors. In the three-factor solution, the broad externalizing factor split into narrower neurodevelopmental (inattention, executive function, and hyperactivity/impulsivity) and social maladjustment factors (aggression, conduct problems, low prosocial behavior, and peer relations, with a lower cross-loading for hyperactivity/impulsivity). The latter factor was moderately associated with the more general broad internalizing factor. The broad internalizing factor was fully represented by a specific internalizing factor in the three-factor solution. This encompassed symptoms of generalized anxiety, panic and obsessive–compulsive disorders, social phobia, separation anxiety, and depression. Symptoms of social phobia were additionally, negatively and more weakly, correlated with the broader social maladjustment factor.

As the sample included more boys than girls, sex differences were explored by comparing boys and girls across the factors (see Table 3). Significant sex differences were observed on the broad externalizing and social maladjustment factors, with boys expressing greater difficulties on both dimensions.[Table tbl3]

### Predictive Value

**Figure 3:**
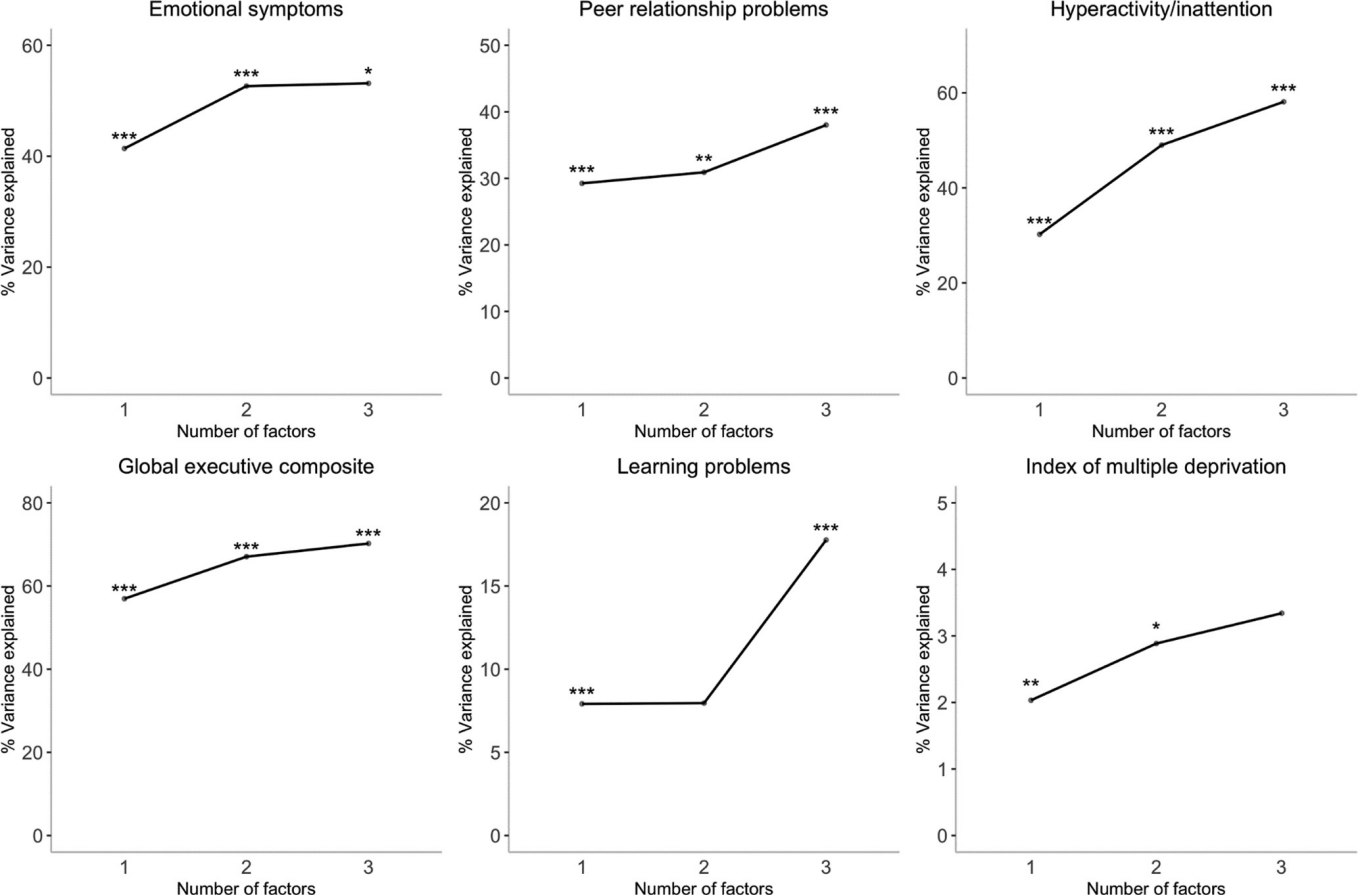
Proportion of Variance Explained in Each Predictor (Adjusted R^2^) by a Given Factor Structure (1- to 3-Factor Solutions) *Note.* Asterisks reflect a significant change in adjusted *R*^2^ for a given structure compared with the simpler structure. * *p* < .05. ** *p* < .01. *** *p* < .001.

A series of linear regressions were performed for each of the following predictors: Emotional symptoms, Peer relationship problems, Hyperactivity and Inattention, Global executive function, Learning Problems, and IMD. For each outcome, three separate regression models were calculated. In the first, factor scores from the one factor model were entered as predictors. In the second, factor scores from both factors in the two-factor model were entered, and in the final model, the three factor scores for the three-factor models were entered. The contributions of the different factor models to each validator were compared using change in *R*^2^ and *F.* The models were compared in pairs (Model 1 vs. Model 2, and Model 2 vs. Model 3) to test whether there was a significant change for a more complex versus a simpler structure. The results are summarized below and shown in Figure 3.

#### Emotional Symptoms

The one, two and three factor models significantly predicted emotional symptoms (Supplemental Materials Table 1). The p factor explained 42% of variance, *F*(1, 380) = 270, *p* < .001. When both factors from the two factor model were entered, 53% of variance was explained, *F*(2, 379) = 212.8, *p* < .001, but only the broad internalizing factor was a significant predictor. The specific internalizing, social maladjustment and neurodevelopmental factors from the three-factor model were significant predictors, explaining 54% of variance, *F*(3, 378) = 145.1, *p* < .001. The addition of more differentiated factors significantly increased the amount of variance explained: the two-factor model accounted for significantly more variance than the one-factor model, Δ*F*(1, 379) = 91.34, *p* < .001, Δ*R*^2^ = .11, and the three factor more than the two factor model, Δ*F*(1, 378) = 5.06, *p* = .025, Δ*R*^2^ = .01. The more complex two-factor structure explained 11% more variance than p-alone, but the change from the two to three factor models was minimal (.6%), despite being significant.

#### Peer Relationship Problems

All factor models significantly predicted peer problems (Supplemental Materials Table 2). The p factor explained 29% of variance, *F*(1, 380) = 158.6, *p* < .001. Both the broad internalizing and broad externalizing factors from the two factor solution were significant predictors, accounting for 31% of variance, *F*(2, 379) = 86.24, *p* < .001. Only the specific internalizing and social maladjustment dimensions from the three-factor solution were significant predictors; the model explained 39% variance, *F*(3, 378) = 78.92, *p* < .001. Adding complexity significantly increased the variance explained. The two-factor model accounted for significantly but marginally (2%) more variance compared with the one-factor model, Δ*F*(1, 379) = 10.12, *p* = .002, Δ*R*^2^ = .02. The three-factor model accounted for significantly more variance (7%) compared with the two-factor model, Δ*F*(1, 378) = 44.48, *p* < .001, Δ*R*^2^ = .07.

#### Hyperactivity and Inattention

All three models significantly predicted symptoms of hyperactivity and inattention (Supplemental Materials Table 3). The p factor alone accounted for 30% of variance, *F*(1, 380) = 166, *p* < .001. The two factor solution explained 49% of variance, *F*(2, 379) = 184.1, *p* < .001, but only the broad externalizing factor was significant. The social maladjustment and neurodevelopmental factors from the three-factor solution were significant predictors; the model explained 59% of variance, *F*(3, 378) = 177.3, *p* < .001. The two-factor model explained significantly more variance than the one factor model Δ*F*(1, 379) = 141.04, *p* < .001, Δ*R*^2^ = .19, and the three factor explained significantly more variance than the two- factor model Δ*F*(1, 378) = 83.45, *p* < .001, Δ*R*^2^ = .09. The variance explained increased substantially with complexity; an increase of 19% from one to two factors, and 10% from two to three factors.

#### Global Executive Function

Each factor solution significantly predicted global executive function problems (Supplemental Materials Table 4). The p factor explained 57% of variance, *F*(1, 377) = 500.8, *p* < .001. The two-factor model accounted for 67% of variance, *F*(2, 376) = 385.8, *p* < .001, and both the broad internalizing and broad externalizing factors were significant. All three dimensions from the three-factor model were significant predictors, explaining 71% of variance, *F*(3, 375) = 298.3, *p* < .001. The two-factor model explained significantly more variance than the one- factor model Δ*F*(1, 376) = 116.87, *p* < .001, Δ*R*^2^ = .10, with an increase of 10% variance explained. The three-factor model explained significantly more variance than the two-factor model Δ*F*(1, 375) = 41.06, *p* < .001, Δ*R*^2^ = .03, but the increase was small (3%).

#### Learning Problems

The one, two and three factor models significantly predicted learning problems (Supplemental Materials Table 5). The p factor explained 8% of variance *F*(1, 379) = 33.66, *p* < .001. The broad internalizing and broad externalizing factors from the two-factor model also explained 8% of variance, *F*(2, 378) = 17.44, *p* < .001. The change in variance explained between these models was not significant, Δ*F*(1, 378) = 1.19, *p* = .276, Δ*R*^2^ = .003. Both the specific internalizing and neurodevelopmental dimensions from the three-factor model significantly predicted learning problems; this model explained 18% of variance, *F*(3, 377) = 28.37, *p* < .001. The specific internalizing and neurodevelopmental factors explained significantly more variance than both the broad internalizing and externalizing factors, Δ*F*(1, 377) = 46.07, *p* < .001, Δ*R*^2^ = .10.

The Learning Problems subscale did not overlap with any of the measures entered into the hierarchical model, but was held out of the primary model so it could be used as an outcome measure. Additional analyses were conducted including the Learning Problems subscale in the model rather than as an outcome. The outcomes of these analyses are reported in Supplemental Materials Table 6. The dimensions that emerged were identical to those produced when Learning Problems was not included, and the Learning Problems subscale loaded on to the neurodevelopmental dimension. This suggests Learning Problems are closely related to the neurodevelopmental dimension: they could be considered indicators of the neurodevelopmental dimension if they are included in the model, or predicted by indicators of this dimension if not.

#### Index of Multiple Deprivation (IMD)

All factor models significantly negatively predicted the IMD (Supplemental Materials Table 7). The single factor model accounted for 2% of variance, *F*(1, 363) = 8.56, *p* = .004. The two-factor model accounted for 3% of variance, *F*(2, 362) = 6.41, *p* = .002, but only the broad externalizing factor was significant. The three-factor model predicted IMD scores *F*(3, 361) = 5.19, *p* = .002, accounting for 4% of the variance, but only the social maladjustment dimension was significant. The two-factor model explained significantly more variance than the one-factor model, Δ*F*(1, 362) = 4.19, *p* = .04, Δ*R*^2^ = .01. The three-factor model did not explain significantly more variance than the two-factor model, Δ*F*(1, 361) = 2.70, *p* = .10, Δ*R*^2^ = .007.

## Discussion

This study provides the first examination of the hierarchical structure of psychopathology in a transdiagnostic sample of children and adolescents with learning-related problems. Six spectra were derived across three levels. The single factor extracted at the apex resembled the p factor, which is common to most dimensional models (Caspi et al., 2014; Kotov et al., 2017; McElroy et al., 2018; Michelini et al., 2019). This captured variance across all the indicators, reflecting a general susceptibility to psychopathology. This single factor predicted all clinical, educational and social outcomes, but the more granular dimensions explained additional variance in most outcomes: this supports the value of extracting multiple higher-order dimensions of psychopathology. At the second level, two broad spectra emerged, internalizing and externalizing. At the third and final level of extraction, three factors capturing internalizing symptoms, social maladjustment and neurodevelopmental problems emerged. The neurodevelopmental dimension proved the strongest predictor of learning problems, underscoring the importance of using hierarchical dimensional models to understand how psychopathology affects different aspects of a child’s functioning.

The two spectra that emerged at the second level of the hierarchy, broad externalizing and broad internalizing, align with early dimensional models of child psychopathology (Achenbach, 1966) and subsequent hierarchical models from community populations of typically developing children (McElroy et al., 2018; Michelini et al., 2019) and adolescents (McElroy et al., 2018; Patalay et al., 2015). The differential associations between these factors and the outcomes provides some validation for their separation: the broad internalizing dimension predicted emotional symptoms, while the broad externalizing dimension predicted hyperactive and inattentive behaviors. An internalizing dimension was also derived in the three-factor model. This encompassed symptoms associated with anxiety and depression, and was perfectly correlated with the broad internalizing spectrum. Together these dimensions resolve the diagnostic comorbidity commonly reported between anxiety and depression, highlighting the value of dimensional approaches in psychopathology.

The broad externalizing dimension identified at the second level split into two narrower factors at the third level of the model. These were (a) social maladjustment that originated from both the broad internalizing and broad externalizing spectra, and (b) neurodevelopmental that emerged from the broad externalizing spectrum only. The social maladjustment and neurodevelopmental factors both encompassed symptoms of ADHD, conduct disorder, and antisocial behavior. Previous studies have generally included ADHD as part of an externalizing spectrum (Bloemen et al., 2018). Our data illustrated a more nuanced picture in which hyperactive/impulsive and inattentive symptoms were rooted in a neurodevelopmental dimension. Both this neurodevelopmental dimension and the social maladjustment dimension predicted independent measures of hyperactivity and inattention that were not included in the model. Together they accounted for significantly and substantially more variance in these symptoms than a single broader externalizing dimension. This indicates that while an externalizing spectrum predicts symptoms of inattention and hyperactivity, more specific and lower-order dimensions can be more informative.

These findings demonstrate two advantages of a hierarchical approach toward dimensional traits in mental health. First, identifying higher-order dimensional traits can mitigate assigning multiple diagnoses (Kotov et al., 2017). For example, different diagnoses like ADHD, conduct disorder and antisocial behavior can load on a common externalizing dimension. Second, the heterogeneity observed in classical diagnostic categories can become more interpretable by identifying granular dimensional traits. For example, symptoms of impulsivity/hyperactivity and inattention can accrue from either the neurodevelopmental dimension or the social maladjustment dimension.

The social maladjustment factor included problems with peer relations, low levels of prosocial behaviors, aggression and conduct problems. Social phobia was also negatively associated with this factor, but only weakly. This negative association could reflect a link between relational victimization and social anxiety; that is, children experiencing conduct-related difficulties might appear less sensitive to negative social evaluation (Carragher et al., 2014). Alternatively, an increase in social maladjustment might reduce the day-to-day impact of peer relations; thus, reducing the prevalence of social anxieties. Consistent with this, the social maladjustment factor predicted a child’s difficulties making and sustaining friendships. Together these findings suggest this dimension captures symptoms of social phobia in addition to social skills and antagonistic behavior.

Adult hierarchical models of psychopathology include a dimension related to antisocial behavior (Kotov et al., 2017) that encompasses many of the same symptoms as our social maladjustment factor. Michelini et al.’s (2019) model, derived from a community sample of children using the same bass-ackward method as us, included a dimension labeled detachment, which also captured variance in children’s social skills. The detachment dimension in the community sample was linked exclusively to a broader internalizing dimension, while the social maladjustment factor derived for our struggling learners was associated with both broader internalizing and externalizing dimensions. The link to externalizing symptoms in children with learning-related problems might reflect the impact of poorer language skills: communication difficulties might cause social problems, and simultaneously cause frustration that leads to problem behaviors. Alternatively, differences between the cohorts could reflect differences in the symptoms measured. Michelini et al. (2019) included a broader range of measures, and subsequently detected more factors across more levels. At the final level, where the detachment dimension emerged, the factors were more specific and narrow than in our model. Detachment tapped only social withdrawal, while our broader social maladjustment factor included a broader set of externalizing behaviors related to social problems.

Our neurodevelopmental dimension included inattention, hyperactivity/impulsivity and executive function problems. It resembled a factor derived in Michelini’s community sample of children, which included similar symptoms of hyperactivity and inattention alongside clumsiness and autistic-like traits (Michelini et al., 2019). Our measures did not assess these latter symptoms, explaining why our neurodevelopmental factor did not encompass these elements. We have nonetheless labeled it neurodevelopmental as it included symptoms of ADHD—a neurodevelopmental condition—and executive function difficulties, which may be part of a neurodevelopmental spectrum alongside symptoms common in ADHD (Holmes et al., 2014). The identification of this dimension in our sample adds to growing evidence for the inclusion of a neurodevelopmental factor in future hierarchical models of both child and adult psychopathology (Michelini et al., 2019).

The predictive value of the derived factor models for learning supports the application of hierarchical dimensional models for determining children’s functioning. The one-factor and the two-factor models predicted learning problems, but each accounted for a small proportion of the variance. The narrower and more specific three-factor model accounted for considerably more: both the specific internalizing and neurodevelopmental factors were significant predictors. The association between symptoms of internalizing difficulties and children’s learning outcomes is consistent with previous evidence (Arnold et al., 2005; Francis et al., 2019; Maughan & Carroll, 2006; Willcutt et al., 2013; Wu et al., 2014). The strong association between the neurodevelopmental factor and learning problems reflects well-documented links between children’s executive function abilities and academic outcomes (Holmes et al., 2020; Peng et al., 2016; Yeniad et al., 2013), and between attentional skills and learning (Follmer, 2018; Landerl & Kölle, 2009; Rotzer et al., 2009). It also reinforces that behavioral inattention and hyperactivity can interfere with classroom learning (e.g., Spira & Fischel, 2005). Crucially, our model suggests these cognitive and affective behaviors may interact to influence learning outcomes in children who are struggling at school. This is clinically informative since individuals who are struggling academically are likely to benefit from both cognitive and behavioral support programs.

The Index of Multiple Deprivation (Ministry of Housing Communities & Local Government, 2020), a measure of socioeconomic status (SES), was predicted by the p factor, and the broad externalizing and social maladjustment dimensions. These findings corroborate a large body of research indicating that deprivation is associated with characteristics of externalizing problems and social difficulties (e.g., McLaughlin et al., 2012; Reiss, 2013). However, because these dimensions accounted for a very small proportion of variance in SES, factors not measured in the current study are likely better predictors.

The purpose of the present study was to characterize psychopathology in the sample as a whole. However, as there were more boys than girls in the sample, as is typical in neurodevelopmental groups (e.g., Gaub & Carlson, 1997; Werling & Geschwind, 2013), factor scores were compared across sexes. Boys expressed greater difficulties on the broad externalizing and social maladjustment dimensions, which captured symptoms related to overt behaviors including aggression, conduct problems, a lack of prosocial behaviors and difficulties with peer relationships. There were no other sex differences. The expression of greater externalizing difficulties in the boys could reflect genuine differences in the manifestations of psychopathology between boys and girls, or broader issues related to socially constructed gender biased or stereotypical views of boys as being disruptive (e.g., Dhuey & Lipscomb, 2010; Hiller et al., 2016; Mowlem et al., 2019). It is not possible to delineate these two possibilities based on the current data.

### Implications for Practice

Category-based diagnostic systems that have thus far guided research and practice have been increasingly criticized (Dalgleish et al., 2020), and clinicians often eschew disorder-specific intervention programs for more eclectic approaches that address multiple difficulties. These emergent programs are comprised of therapeutic packages that address symptoms commonly experienced by individuals irrespective of their official diagnostic status (e.g., Weisz et al., 2012). The current data contribute to these approaches by highlighting symptom dimensions underlying mental ill-health in those with neurodevelopmental conditions. Crucially, they indicate that symptoms observed in youth known to be struggling with learning are organized broadly similarly to those observed in community samples of children (e.g., Michelini et al., 2019). This could imply that the salutary effects of interventions in one population could generalize well to others within that dimension. Findings also indicate that a neurodevelopmental spectrum should be included in dimensional models of psychopathology: in children with learning difficulties, cognitive and affective symptoms might interact along this dimension. This is important as it may help to identify those experiencing learning problems as well as classic internalizing and externalizing difficulties.

Using hierarchical dimensional models in practice is not without its challenges, but they should be adopted when working with children and adolescents with known cognitive difficulties that affect their learning. Guiding principles for practitioners include using ranges of scores along dimensions of spectra, and not cut-offs that define the “presence” or “absence” of a condition. This approach has been taken in some areas of medicine, where data-driven categories are imposed on dimensional measures (e.g., normal, mild, moderate, and severe blood pressure). Dimensional approaches can also help clinicians adopt an efficacious approach to treatment. By focusing interventions on higher-order spectra (e.g., social maladjustment), it could be possible to efficiently address the core components that underlie a range of idiosyncratic and lower-order symptom complaints (i.e., impulsivity/hyperactivity, conduct symptoms, and antisocial behavior). These approaches could provide useful adjuncts to extant intervention approaches and might provide a heuristic framework that reflects what already happens in clinical practice.

### Limitations and Future Directions

The primary limitation of this study is that it included only a limited range of assessments of psychopathology and neurodevelopmental difficulties, provided by a parent/carer/guardian. Most notably there was a lack of assessments related to symptoms of autism. This was unavoidable due to the restricted set of measures administered in the cohort protocol (see Holmes et al., 2019). Related to this, using subscale scores rather than symptom-level data may have masked some of the heterogeneity within the subscales (see Forbes et al., 2021, for a full discussion of the issues around using composite scores in dimensional models). Different symptoms defining the subscale scores may have aligned with different dimensions to other symptoms contributing to the same subscale (e.g., some symptoms of generalized anxiety disorder may have loaded on an internalizing spectrum, and others on the neurodevelopmental dimension). Nonetheless, key dimensions replicated previous studies using different measures and methods with typical samples, providing validity to our model (e.g., Kotov et al., 2017; Michelini et al., 2019). Replication with a more comprehensive set of measures, and with symptom-level ratings from multiple informers, would be one way to validate and extend the current model. Some argue that dimensional models are statistical artefacts (see Basilevsky, 2009; Mulaik, 1986). However, the predictive utility of the derived dimensions suggest otherwise. Future research is needed to guide the use of hierarchical dimensional models in clinical training and practice, and to elucidate their etiology and biomarkers.

## Supplementary Material

10.1037/abn0000710.supp

## Figures and Tables

**Table 1 tbl1:** Descriptive Statistics

		Raw scores				*t*-scores				% in clinical/abnormal range
Measure^a,b^	*N*	*M*	*SD*	Min	Max	*M*	*SD*	Min	Max	
RCADS										
Depression	401	9.10	5.21	0	27	65.18	12.87	37	81	43.53
Generalized anxiety disorder	398	5.98	3.97	0	18	56.54	12.47	36	81	19.05
Panic disorder	396	3.91	4.12	0	27	57.44	13.64	40	81	23.93
Social phobia	400	12.44	6.34	0	27	59.55	14.18	29	81	26.18
Separation anxiety	402	6.86	5.05	0	21	59.45	14.94	36	81	28.29
Obsessive-compulsive disorder	399	3.01	3.09	0	15	53.74	11.16	41	81	11.25
CPSF										
Aggression	400	4.01	4.18	0	15	66.43	17.65	44	91	42.11
Hyperactivity/impulsivity	402	11.44	5.33	0	18	77.08	14.78	41	90	67.58
Executive function	400	10.62	3.35	0	15	76.06	11.62	40	90	74.94
Inattention	402	12.06	3.18	0	15	82.09	10.11	44	90	87
Peer relations	398	5.88	4.40	0	15	75.05	16.90	44	90	63.98
Learning problems	401	9.72	3.64	0	15	75.97	12.52	41	90	70.75
SDQ										
Conduct problems	401	3.78	2.61	0	10	—	—	—	—	51.86
Prosocial behavior	401	6.55	2.39	0	10	—	—	—	—	46.4
Emotional symptoms	401	4.48	2.78	0	10	—	—	—	—	49.13
Peer relationship problems	401	3.62	2.53	0	10	—	—	—	—	48.14
Hyperactivity/inattention	401	7.87	2.25	1	10	—	—	—	—	64.02
Global executive composite (BRIEF)	396	169.88	25.64	84	215	72.81	10.85	35	98	67.93
Index of multiple deprivation	386	19,897.54	8,538.58	155	32,803	—	—	—	—	—
*Note*. SDQ = Strengths and Difficulties Questionnaire; CPSF = Conners-3 Parent Rating Scale Short Form; RCADS = Revised Child and Anxiety and Depression Scale (Parent Version); BRIEF = The Behavior Rating Inventory of Executive Function.										
^a^ For RCADS, CPSF, SDQ, and BRIEF subscales higher raw and *T*-scores indicate greater difficulties. SDQ Prosocial behavior is an exception—higher scores indicate greater strengths. The index of multiple deprivation ranks areas in England from the most to least deprived. Lower indices reflect greater deprivation and higher indices reflect less deprivation. ^b^ Clinical levels: *T*-score of 70 or above for all RCADS, CPSF, and BRIEF subscales; raw score equal or higher than 5 for SDQ Emotional symptoms; raw score equal or higher than 4 for SDQ Conduct problems and Peer relationships problems; raw score equal or higher than 7 for SDQ Hyperactivity/inattention; and raw score equal or lower than 5 for SDQ Prosocial behavior.										

**Table 2 tbl2:** Factor Loadings for Rotated (Geomin) Solutions Extracted Using Principal Components

	One-factor	Two-factors		Three-factors		
Measure	P	Broad internalizing	Broad externalizing	Specific internalizing	Social maladjustment	Neuro developmental
RCADS						
Depression	**0.84**	**0.67**	0.34	**0.69**	0.23	0.12
Generalized anxiety disorder	**0.75**	**0.87**	0.01	**0.9**	−0.06	0
Panic disorder	**0.69**	**0.84**	−0.02	**0.86**	0.01	−0.13
Social phobia	**0.52**	**0.83**	−0.23	**0.86**	**−0.39**	0.07
Separation anxiety	**0.72**	**0.79**	0.06	**0.81**	0.04	−0.05
Obsessive-compulsive disorder	**0.68**	**0.77**	0.04	**0.78**	0.07	−0.1
CPSF						
Aggression	**0.7**	0.13	**0.73**	0.14	**0.83**	−0.01
Hyperactivity/impulsivity	**0.6**	−0.04	**0.81**	−0.02	**0.4**	**0.62**
Executive function	**0.54**	0.06	**0.61**	0.1	−0.02	**0.84**
Inattention	**0.51**	−0.04	**0.67**	0	0.02	**0.89**
Peer relations	**0.57**	0.22	**0.49**	0.22	**0.54**	−0.01
SDQ						
Conduct problems	**0.64**	0	**0.8**	0.01	**0.81**	0.11
Prosocial behavior	**0.54**	−0.03	**0.71**	−0.03	**0.78**	0.03
Eigenvalues	5.41	3.97	3.66	4.11	2.75	2.05
% of variance	41.63	30.51	28.15	31.65	21.16	15.78
Factor correlations						
P						
Broad internalizing	0.85					
Broad externalizing	0.8	0.37				
Specific internalizing	0.88	1	0.42			
Social maladjustment	0.7	0.32	0.88	0.36		
Neurodevelopmental	0.56	0.24	0.72	0.3	0.3	
*Note*. SDQ = Strengths and Difficulties Questionnaire; CPSF = Conners-3 Parent Rating Scale Short Form; RCADS = Revised Child and Anxiety and Depression Scale (Parent Version). Factor correlations are presented in the bottom panel. Loadings above .35 are presented in bold. All factor correlations were statistically significant (*p* < .001).										

**Table 3 tbl3:** Factor Score Descriptive Statistics for Boys and Girls Together With Test Statistics

	Boys			Girls					
Models	*N*	*M*	*SD*	*N*	*M*	*SD*	*t*	*p*	Cohen’s *d*
One-factor									
P	266	0.018	0.975	116	−0.091	1.060	0.946	.345	−0.109
Two-factor									
Broad internalizing	266	−0.039	0.973	116	0.045	1.039	−0.740	.460	0.085
Broad externalizing	266	0.077	0.964	116	−0.211	1.061	2.504	.013	−0.290
Three-factor									
Internalizing	266	−0.032	0.975	116	0.028	1.042	−0.531	.596	0.061
Social maladjustment	266	0.068	0.974	116	−0.181	1.062	2.161	.032	−0.249
Neurodevelopmental	266	0.057	0.964	116	−0.164	1.078	1.900	.059	−0.221
