# Supplementary material for: Higher-Order Dimensions of Psychopathology in a Neurodevelopmental Transdiagnostic Sample
Source: J Abnorm Psychol. 2021 Nov;130(8):909–22. doi: 10.1037/abn0000710 (PMC8628482; doi:10.1037/abn0000710)
Supplement: Supplementary file 1 [file ABN-2021-2069_Suppl.docx]

**Supplementary Table 1**

*Regression Results Using Emotional Symptoms (SDQ) as the Criterion*

| Predictor | *b* | *b*  95% CI | *beta* | *beta*  95% CI | *r* | Fit | Difference |
| --- | --- | --- | --- | --- | --- | --- | --- |
| (Intercept) | -0.01 | [-0.08, 0.07] |  |  |  |  |  |
| P | 0.65** | [0.57, 0.73] | 0.64 | [0.57, 0.72] | .64** |  |  |
|  |  |  |  |  |  | *R^2^*  = .415** |  |
|  |  |  |  |  |  |  |  |
| (Intercept) | -0.00 | [-0.07, 0.07] |  |  |  |  |  |
| Broad internalising | 0.72** | [0.64, 0.79] | 0.70 | [0.63, 0.78] | .73** |  |  |
| Broad externalising | 0.06 | [-0.02, 0.13] | 0.06 | [-0.02, 0.13] | .32** |  |  |
|  |  |  |  |  |  | *R^2^*  = .529** | Δ*R^2^*  = .114** |
|  |  |  |  |  |  |  |  |
| (Intercept) | -0.01 | [-0.07, 0.06] |  |  |  |  |  |
| Specific internalising | 0.73** | [0.66, 0.81] | 0.72 | [0.65, 0.80] | .73** |  |  |
| Social maladjustment | 0.08* | [0.00, 0.15] | 0.08 | [0.00, 0.15] | .31** |  |  |
| Neurodevelopmental | -0.09* | [-0.16, -0.01] | -0.09 | [-0.16, -0.01] | .16** |  |  |
|  |  |  |  |  |  | *R^2^*  = .535** | Δ*R^2^*  = .006* |
|  |  |  |  |  |  |  |  |

*Note.* A significant *b*-weight indicates the beta-weight is also significant. *b* represents unstandardized regression weights.

*beta* indicates the standardized regression weights. *r* represents the zero-order correlation.

* indicates *p* < .05. ** indicates *p* < .01.

**Supplementary Table 2**

*Regression Results Using Peer Relationship Problems (SDQ) as the Criterion*

| Predictor | *b* | *b*  95% CI | *beta* | *beta*  95% CI | *r* | Fit | Difference |
| --- | --- | --- | --- | --- | --- | --- | --- |
| (Intercept) | -0.00 | [-0.09, 0.08] |  |  |  |  |  |
| P | 0.54** | [0.46, 0.63] | 0.54 | [0.46, 0.63] | .54** |  |  |
|  |  |  |  |  |  | *R^2^*  = .294** |  |
|  |  |  |  |  |  |  |  |
| (Intercept) | -0.00 | [-0.09, 0.08] |  |  |  |  |  |
| Broad internalising | 0.23** | [0.14, 0.32] | 0.23 | [0.14, 0.32] | .39** |  |  |
| Broad externalising | 0.43** | [0.34, 0.52] | 0.43 | [0.34, 0.52] | .52** |  |  |
|  |  |  |  |  |  | *R^2^*  = .313** | Δ*R^2^*  = .018** |
|  |  |  |  |  |  |  |  |
| (Intercept) | -0.01 | [-0.08, 0.07] |  |  |  |  |  |
| Specific internalising | 0.24** | [0.15, 0.33] | 0.24 | [0.15, 0.33] | .41** |  |  |
| Social maladjustment | 0.51** | [0.42, 0.59] | 0.51 | [0.42, 0.60] | .58** |  |  |
| Neurodevelopmental | -0.05 | [-0.13, 0.04] | -0.05 | [-0.13, 0.04] | .18** |  |  |
|  |  |  |  |  |  | *R^2^*  = .385** | Δ*R^2^*  = .072** |
|  |  |  |  |  |  |  |  |

*Note.* A significant *b*-weight indicates the beta-weight is also significant. *b* represents unstandardized regression weights.

*beta* indicates the standardized regression weights. *r* represents the zero-order correlation.

* indicates *p* < .05. ** indicates *p* < .01.

**Supplementary Table 3**

*Regression Results Using Hyperactivity/Inattention (SDQ) as the Criterion*

| Predictor | *b* | *b*  95% CI | *beta* | *beta*  95% CI | *r* | Fit | Difference |
| --- | --- | --- | --- | --- | --- | --- | --- |
| (Intercept) | 0.01 | [-0.08, 0.09] |  |  |  |  |  |
| P | 0.55** | [0.47, 0.64] | 0.55 | [0.47, 0.64] | .55** |  |  |
|  |  |  |  |  |  | *R^2^*  = .304** |  |
|  |  |  |  |  |  |  |  |
| (Intercept) | 0.01 | [-0.07, 0.08] |  |  |  |  |  |
| Broad internalising | -0.02 | [-0.10, 0.06] | -0.02 | [-0.10, 0.06] | .24** |  |  |
| Broad externalising | 0.71** | [0.63, 0.79] | 0.71 | [0.63, 0.79] | .70** |  |  |
|  |  |  |  |  |  | *R^2^*  = .493** | Δ*R^2^*  = .189** |
|  |  |  |  |  |  |  |  |
| (Intercept) | 0.01 | [-0.06, 0.07] |  |  |  |  |  |
| Specific internalising | -0.00 | [-0.08, 0.07] | -0.00 | [-0.08, 0.07] | .29** |  |  |
| Social maladjustment | 0.29** | [0.21, 0.36] | 0.29 | [0.22, 0.36] | .47** |  |  |
| Neurodevelopmental | 0.63** | [0.56, 0.70] | 0.63 | [0.56, 0.70] | .71** |  |  |
|  |  |  |  |  |  | *R^2^*  = .585** | Δ*R^2^*  = .092** |
|  |  |  |  |  |  |  |  |

*Note.* A significant *b*-weight indicates the beta-weight is also significant. *b* represents unstandardized regression weights.

*beta* indicates the standardized regression weights. *r* represents the zero-order correlation

* indicates *p* < .05. ** indicates *p* < .01.

**Supplementary Table 4**

*Regression Results Using Global Executive Function Composite (BRIEF) as the Criterion*

| Predictor | *b* | *b*  95% CI | *beta* | *beta*  95% CI | *r* | Fit | Difference |
| --- | --- | --- | --- | --- | --- | --- | --- |
| (Intercept) | 72.83** | [72.11, 73.55] |  |  |  |  |  |
| P | 8.18** | [7.46, 8.90] | 0.76 | [0.69, 0.82] | .76** |  |  |
|  |  |  |  |  |  | *R^2^*  = .571** |  |
|  |  |  |  |  |  |  |  |
| (Intercept) | 72.81** | [72.18, 73.44] |  |  |  |  |  |
| Broad internalising | 2.28** | [1.59, 2.96] | 0.21 | [0.15, 0.27] | .47** |  |  |
| Broad externalising | 7.78** | [7.11, 8.46] | 0.72 | [0.66, 0.78] | .80** |  |  |
|  |  |  |  |  |  | *R^2^*  = .672** | Δ*R^2^*  = .102** |
|  |  |  |  |  |  |  |  |
| (Intercept) | 72.82** | [72.22, 73.42] |  |  |  |  |  |
| Specific internalising | 2.53** | [1.87, 3.19] | 0.23 | [0.17, 0.29] | .52** |  |  |
| Social maladjustment | 4.08** | [3.43, 4.73] | 0.38 | [0.32, 0.44] | .61** |  |  |
| Neurodevelopmental | 5.48** | [4.84, 6.11] | 0.51 | [0.45, 0.57] | .69** |  |  |
|  |  |  |  |  |  | *R^2^*  = .705** | Δ*R^2^*  = .032** |
|  |  |  |  |  |  |  |  |

*Note.* A significant *b*-weight indicates the beta-weight is also significant. *b* represents unstandardized regression weights.

*beta* indicates the standardized regression weights. *r* represents the zero-order correlation.

* indicates *p* < .05. ** indicates *p* < .01.

**Supplementary Table 5**

*Regression Results Using Learning Problems (CPSF) as the Criterion*

| Predictor | *b* | *b*  95% CI | *beta* | *beta*  95% CI | *r* | Fit | Difference |
| --- | --- | --- | --- | --- | --- | --- | --- |
| (Intercept) | 75.81** | [74.59, 77.04] |  |  |  |  |  |
| P | 3.61** | [2.38, 4.83] | 0.29 | [0.19, 0.38] | .29** |  |  |
|  |  |  |  |  |  | *R^2^*  = .082** |  |
|  |  |  |  |  |  |  |  |
| (Intercept) | 75.81** | [74.58, 77.03] |  |  |  |  |  |
| Broad internalising | 1.74* | [0.41, 3.07] | 0.14 | [0.03, 0.24] | .22** |  |  |
| Broad externalising | 2.67** | [1.35, 4.00] | 0.21 | [0.11, 0.31] | .26** |  |  |
|  |  |  |  |  |  | *R^2^*  = .084** | Δ*R^2^*  = .003 |
|  |  |  |  |  |  |  |  |
| (Intercept) | 75.79** | [74.64, 76.95] |  |  |  |  |  |
| Specific internalising | 1.85** | [0.57, 3.13] | 0.15 | [0.04, 0.25] | .24** |  |  |
| Social maladjustment | -1.08 | [-2.34, 0.18] | -0.09 | [-0.19, 0.01] | .08 |  |  |
| Neurodevelopmental | 4.95** | [3.68, 6.21] | 0.39 | [0.29, 0.48] | .41** |  |  |
|  |  |  |  |  |  | *R^2^*  = .184** | Δ*R^2^*  = .100** |
|  |  |  |  |  |  |  |  |

*Note.* A significant *b*-weight indicates the beta-weight is also significant. *b* represents unstandardized regression weights.

*beta* indicates the standardized regression weights. *r* represents the zero-order correlation.

* indicates *p* < .05. ** indicates *p* < .01.

**Supplementary Table 6**

*Factor Loadings for Rotated (geomin) Solutions Extracted Using Principal Components including CPSF Learning Problems scale*

|  | 1-factor | 2-factors | | 3-factors | | |
| --- | --- | --- | --- | --- | --- | --- |
|  | P | Broad internalising | Broad externalising | Specific internalising | Social maladjustment | Neurodevelopmental |
| RCADS |  |  |  |  |  |  |
| Depression | **0.83** | **0.67** | **0.34** | **0.69** | 0.25 | 0.1 |
| Generalised anxiety disorder | **0.74** | **0.87** | 0.01 | **0.89** | -0.02 | -0.04 |
| Panic disorder | **0.69** | **0.84** | -0.03 | **0.86** | 0 | -0.11 |
| Social phobia | **0.52** | **0.82** | -0.21 | **0.83** | **-0.39** | 0.1 |
| Separation anxiety | **0.71** | **0.79** | 0.06 | **0.81** | 0.04 | -0.03 |
| Obsessive-compulsive disorder | **0.68** | **0.77** | 0.04 | **0.79** | 0.06 | -0.08 |
| CPSF |  |  |  |  |  |  |
| Aggression | **0.69** | 0.14 | **0.71** | 0.18 | **0.79** | 0.01 |
| Hyperactivity/impulsivity | **0.61** | -0.05 | **0.81** | -0.04 | **0.46** | **0.54** |
| Executive function | **0.56** | 0.04 | **0.65** | 0.04 | 0.02 | **0.83** |
| Inattention | **0.53** | -0.05 | **0.71** | -0.05 | 0.08 | **0.85** |
| Learning problems | **0.35** | 0.11 | 0.33 | 0.09 | -0.27 | **0.73** |
| Peer relations | **0.57** | 0.22 | **0.47** | 0.25 | **0.53** | -0.01 |
| SDQ |  |  |  |  |  |  |
| Conduct problems | **0.64** | 0.01 | **0.78** | 0.04 | **0.8** | 0.1 |
| Prosocial behaviour | **0.53** | -0.02 | **0.68** | 0.01 | **0.75** | 0.03 |
| Eigenvalues | 5.52 | 3.98 | 3.76 | 4.13 | 2.77 | 2.35 |
| % of variance | 39.4 | 28.45 | 26.83 | 29.5 | 19.76 | 16.79 |
| *Factor correlations* |  |  |  |  |  |  |
| P |  |  |  |  |  |  |
| Broad internalising | 0.84 |  |  |  |  |  |
| Broad externalising | 0.81 | 0.37 |  |  |  |  |
| Specific internalising | 0.87 | 1 | 0.42 |  |  |  |
| Social maladjustment | 0.67 | 0.28 | 0.86 | 0.32 |  |  |
| Neurodevelopmental | 0.62 | 0.29 | 0.75 | 0.34 | 0.31 |  |

*Note*. Factor correlations are presented in the bottom panel. Loadings above 0.35 presented in bold. All factor correlations were statistically significant (*p* < 0.001). SDQ = Strengths and Difficulties Questionnaire; CPSF = Conners-3 Parent Rating Scale Short Form; RCADS = Revised Child and Anxiety and Depression Scale (Parent Version).

**Supplementary Table 7**

*Regression Results Using the Index of Multiple Deprivation as the Criterion*

| Predictor | *b* | *b*  95% CI | *beta* | *beta*  95% CI | *r* | Fit | Difference |
| --- | --- | --- | --- | --- | --- | --- | --- |
| (Intercept) | 19817.16** | [18944.29, 20690.02] |  |  |  |  |  |
| P | -1319.80** | [-2207.18, -432.42] | -0.15 | [-0.25, -0.05] | -.15** |  |  |
|  |  |  |  |  |  | *R^2^*  = .023** |  |
|  |  |  |  |  |  |  |  |
| (Intercept) | 19836.42** | [18967.16, 20705.67] |  |  |  |  |  |
| Broad internalising | -65.33 | [-1013.37, 882.71] | -0.01 | [-0.12, 0.10] | -.07 |  |  |
| Broad externalising | -1571.64** | [-2507.91, -635.37] | -0.18 | [-0.29, -0.07] | -.18** |  |  |
|  |  |  |  |  |  | *R^2^*  = .034** | Δ*R^2^*  = .011* |
|  |  |  |  |  |  |  |  |
| (Intercept) | 19832.67** | [18965.43, 20699.91] |  |  |  |  |  |
| Specific internalising | -87.93 | [-1053.30, 877.44] | -0.01 | [-0.12, 0.10] | -.08 |  |  |
| Social maladjustment | -1665.34** | [-2601.66, -729.01] | -0.20 | [-0.31, -0.09] | -.20** |  |  |
| Neurodevelopmental | -105.73 | [-1035.41, 823.96] | -0.01 | [-0.12, 0.10] | -.07 |  |  |
|  |  |  |  |  |  | *R^2^*  = .041** | Δ*R^2^*  = .007 |
|  |  |  |  |  |  |  |  |

*Note.* A significant *b*-weight indicates the beta-weight is also significant. *b* represents unstandardized regression weights.

*beta* indicates the standardized regression weights. *r* represents the zero-order correlation.

* indicates *p* < .05. ** indicates *p* < .01.

**Supplementary Table 8**

*Factor Loadings for Rotated (geomin) Solution with Four Factors Extracted Using Principal Components*

|  | Factor 1 | Factor 2 | Factor 3 | Factor 4 |
| --- | --- | --- | --- | --- |
| RCADS |  |  |  |  |
| Depression | **0.65** | 0.16 | 0.13 | 0.23 |
| Generalised anxiety disorder | **0.91** | -0.07 | 0.03 | -0.07 |
| Panic disorder | **0.89** | 0 | -0.09 | -0.12 |
| Social phobia | **0.82** | **-0.44** | 0.07 | 0.09 |
| Separation anxiety | **0.79** | -0.02 | -0.03 | 0.12 |
| Obsessive-compulsive disorder | **0.85** | 0.11 | -0.05 | -0.26 |
| CPSF |  |  |  |  |
| Aggression | 0.16 | **0.82** | 0.02 | 0.04 |
| Hyperactivity/impulsivity | 0.01 | **0.49** | **0.64** | -0.12 |
| Executive function | 0.01 | -0.05 | **0.81** | 0.32 |
| Inattention | -0.02 | 0.09 | **0.88** | 0.01 |
| Peer relations | 0.09 | 0.33 | -0.05 | **0.73** |
| Aggression | 0.16 | **0.82** | 0.02 | 0.04 |
| SDQ |  |  |  |  |
| Conduct problems | 0.03 | **0.82** | 0.13 | 0.05 |
| Prosocial behaviour | -0.09 | **0.67** | 0.01 | **0.41** |
| Eigenvalues | 4.06 | 2.51 | 2.02 | 1.07 |
| % of variance | 31 | 19 | 16 | 8 |
| Factor 1 | - | - | - | - |
| Factor 2 | 0.34** | - | - | - |
| Factor 3 | 0.28** | 0.21** | - | - |
| Factor 4 | 0.29** | 0.13* | 0.14* | - |

*Note*. Factor correlations are presented in the bottom panel. Loadings above 0.35 presented in bold. SDQ = Strengths and Difficulties Questionnaire; CPSF = Conners-3 Parent Rating Scale Short Form; RCADS = Revised Child and Anxiety and Depression Scale (Parent Version).

***p* <.001; * *p* <.01
